# Characterization of COVID-19 outbreaks in three nursing homes during the first wave in Berlin, Germany

**DOI:** 10.1038/s41598-021-04115-9

**Published:** 2021-12-24

**Authors:** Alexandra Roth, Silke Feller, Andreas Ruhnau, Lena Plamp, Ute Viereck, Kerstin Weber, Dominic Maertens, Ilona Hoor, Ronny Gamradt, Pia Freyer, Frank Wenke-Gellert, Andreas Terjaew, Andreas Zintel, Juliane Markus, Ines Gögelein-Mahfouz, Nicolai Savaskan

**Affiliations:** 1 Department of Public Health Neukölln, District Office Neukölln of Berlin Neukölln, Blaschkoallee 32, 12359 Berlin, Germany; 2 University of Applied Sciences, Fulda, Germany; 3 Department of Legal Office Neukölln, District Office of Berlin Neukölln, Berlin, Germany

**Keywords:** Diseases, Infectious diseases, Viral infection, Health care, Public health

## Abstract

Severe Acute Respiratory Syndrome Coronavirus 2 (SARS-CoV-2) belongs to the coronavirus family and is characterized by its high transmission competence. Elderly COVID-19 patients are at significantly higher risk of severe course of disease and death. Therefore, outbreaks in nursing homes are particularly challenging for facility managers and health authorities. Here, we report three outbreaks of COVID-19 related to nursing homes (NH01.a, NH02 and NH03) with almost 1000 affected individuals during the first COVID-19 wave in Berlin, Germany. The occurrence of cases and the measures taken were analyzed retrospectively. In all three outbreaks, the index persons were nursing home employees or volunteers. Measures taken were quarantine of contacts, close-meshed tests, separation of the affected housing unit, suspension of admission, ban on visiting, and equipping staff with personal protective equipment, of which there was a shortage in Germany at the beginning of the pandemic. A court-ordered quarantine became necessary for three residents of NH01.a due to cognitive disabilities. In total, 61 persons were tested positive for SARS-CoV-2 in NH01.a, ten persons in NH02, and sixteen persons in NH03. Seventeen patients (27.9%) of NH01.a and three patients (18.8%) of NH03 were referred to hospital. Of all confirmed cases, thirteen (21.3%) related to NH01.a and four (25.0%) related to NH03 died as a result of the infection. Besides one 82 year old volunteer, all deceased persons were residents aged between 66 and 98. Our results emphasize the importance of a previously developed containment and cluster strategy for nursing homes. Due to the particular vulnerability of the residents, immediate action, close cooperation and communication between the facility management, residents, visitors and the health authorities are essential in the case of confirmed COVID-19 cases in healthcare facilities.

## Introduction

Cases of infection with the new Severe Acute Respiratory Syndrome Coronavirus 2 (SARS-CoV-2) were first reported on the 31st of December 2019 from Wuhan, Hubei Province, China^1^. Within the following weeks, SARS-CoV-2 spread globally. This development prompted the World Health Organization (WHO) to declare a pandemic on the 11th of March 2020. In Germany, the first COVID-19 case occurred at the end of January 2020^2^. During the second week of March, first cases became apparent in Berlin, including Neukölln, a highly populated district in the south of Berlin, with a population of nearly 330,000 inhabitants^3^.

Elderly people are at higher risk of severe disease and death^1^. Outbreaks in nursing homes are therefore a major problem worldwide. This was particularly true in the early stages of the pandemic, when knowledge of the spread and treatment options of the disease was still poor, testing capacity limited, and vaccination unavailable. For example, for the first 5 months of the pandemic, Abrams et al. calculated that 42% of all COVID-19 deaths across 38 U.S. states were reported by nursing homes and other long-term care facilities^4^. In Germany, 681 outbreaks in nursing homes resulting in 12,681 cases were reported during the first wave. This corresponds to 14% of all outbreaks and 10% of all cases during this period^5^. Prior to the start of the second wave in fall 2020, 86% of patients in Germany and 82% of patients in Neukölln who had died of COVID-19, were 70 years of age or older^6^. The median age of deceased COVID-19 patients in Germany was 82 years, and in Neukölln 80 years; while the median age of infected persons in Germany was 45 years, and in Neukölln 35 years^7^. Germany is one of the countries with the oldest age structure in the world, putting a large proportion of the population at particular risk^8^. The age group of 70 and above represents approximately 16% in Germany and 13% in Neukölln^3,9^. Accordingly, it is essential to investigate outbreaks in nursing homes in detail in order to develop and implement appropriate protective measures.

In Neukölln, there are twenty-three nursing homes, providing care for up to 2285 senior citizens, with about 1400 employees working in these facilities^10^. Pre-existing conditions such as heart and lung diseases, diabetes, and cancer are very common among residents of nursing homes, and are also considered to be risk factors for severe cases of COVID-19^1^. Once transmitted into a nursing home, specific conditions of accommodation, community activities and care promote rapid spread of the virus. In the early stage of the first wave of the pandemic, there was also a lack of personal protective equipment (PPE) for nursing and medical staff in Germany.

**Figure 1:**
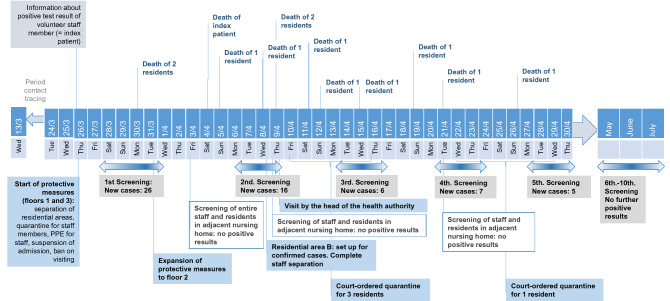
Timeline of events and directed measures in nursing home NH01.a. Graphical representation of the chronological sequence of screenings, new infections, deaths and measures taken to contain the outbreak.

**Figure 2:**
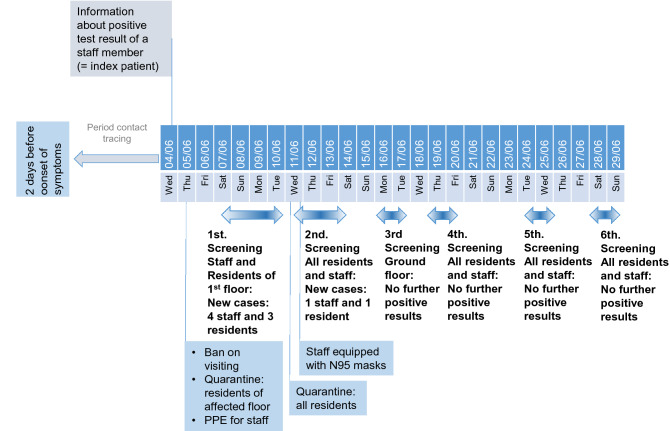
Timeline of events and directed measures in nursing home NH02. Graphical representation of the chronological sequence of screenings, new infections, deaths and measures taken to contain the outbreak.

**Figure 3:**
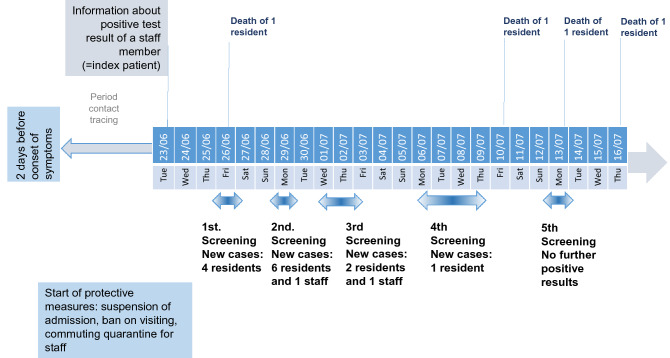
Timeline of events and directed measures in nursing home NH03. Graphical representation of the chronological sequence of screenings, new infections, deaths and measures taken to contain the outbreak.

Here, we report COVID-19 outbreaks in three nursing homes in Neukölln in March and April 2020 (NH01.a) and in June and July 2020 (NH02 and NH03) (Figs. 1, 2, 3). A special aspect of NH01.a, a nursing home for 93 elderly persons, is its organizational and physical connection to another nursing home (NH01.b), where up to one hundred nineteen senior citizens lived. The objective of this study is to describe and analyze the characteristics of the outbreaks and the measures taken to contain them. The purpose of this paper is to help improve the containment of outbreaks in nursing homes, by lending insight into what went well and what could have been improved in the three cases.

## Material and methods

### Study design and study population

We conducted a retrospective observational study of nursing homes in Neukölln that experienced a COVID-19 outbreak in the first pandemic wave. We used data collected as part of outbreak and pandemic response by the local health authority.

The abovementioned nursing homes provide non-acute long-term care for elderly people of both genders in single or double rooms. The residents are subdivided into living groups of about ten to fifteen people, who share various common areas.

**Figure 4:**
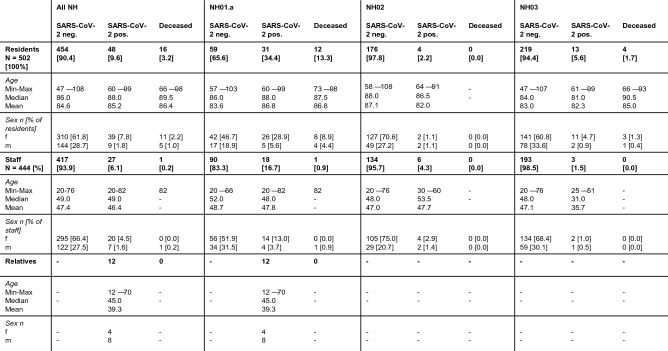
Characteristics of residents, staff members and relatives in three affected COVID-19 affected nursing homes. Data are given for all nursing homes (NH01-03) in summary as well as for each single nursing home. Data are given in (%), mean or median, unless stated otherwise.

The two nursing homes NH01.a and NH01.b are connected without restrictions. Thus, there are numerous social contacts across both houses. For larger events the NH01.a offers an event hall, which is also used by the residents of the NH01.b. In addition, the NH01.a has a largescale kitchen, where meals are prepared for both facilities as well as for two further nursing homes located in other parts of Berlin. NH01.a has a maximum capacity of ninety-three persons. Ninety residents aged 57 to 103 lived there at the time of the outbreak, 75.6% of them were female (Fig. 4). One hundred and eight people were employed in care and service, both permanent and leased staff, including the index person, an 82-year-old volunteer. At the time of the outbreak, 118 people were living at NH01.b. The residents of NH01.b were comparable in age and sex characteristics to those of NH01.a. Eighty people worked there at the beginning of the outbreak, two of them in both nursing homes.

NH02 consists of six floors, which form residential groups of 16 to 36 residents. A total of 180 elderly persons aged 58 to 108 lived in NH02 at the time of the outbreak, 71.7% of them were female. One hundred and forty people were employed there (Fig. 4).

At the time of the outbreak, 232 residents aged 47 to 107 lived in five residential groups in NH03. In total, 196 permanent and leased staff members worked in NH03 (Fig. 4).

### Tasks of the Department of Public Health Neukölln

With rising infection numbers in Germany, the pandemic squad at the Department of Public Health Neukölln was formed in February 2020. It mainly comprised of employees from all areas of local and federal public service. In addition, the pandemic squad Neukölln was also assisted by the German Army (Bundeswehr) and external staff. All positive SARS-CoV-2 cases of residents of Neukölln are reported to the pandemic squad. They also carry out tests themselves, particularly in community facilities such as nursing homes, schools and childcare facilities. Confirmed cases are reported by the pandemic squad as secondary data to the state public health authority (Landesamt für Gesundheit und Soziales) and the governmental Public Health Institution (Robert Koch Institute, RKI). The tasks of the pandemic squad include informing the population, tracing contacts of SARS-CoV-2 positive tested individuals, and ordering measures to contain the infection. All the measures carried out were part of the statutory tasks of Germany’s local departments of public health and were thus legally approved on the basis of Germany’s Infection Protection Act^11^ All measures were carried out according to the regulations and guidelines of the RKI^6,12,13^. The study was exempt from regular institutional review board approval requirements because it was conducted in response to a public health emergency. The Neukölln municipal review board exempted the study approval.

### Laboratory testing

For the diagnosis of SARS-CoV-2 infection, throat swabs of all residents and staff members were taken by a mobile swab team of the pandemic squad on site at the nursing homes. Testing in NH01.a was repeated weekly until no new positive swabs and no symptoms occurred for 9 weeks. After the subsequent outbreaks in NH02 and NH03, residents and staff of all nursing homes in Neukölln were tested weekly to detect outbreaks at an early stage. Samples were taken and transported according to the regulations and guidelines of the Robert Koch Institute, after informed consent was obtained from all subjects or, if necessary, by their legal representatives^12^. Reverse transcription-polymerase chain reaction (RT-PCR) was performed according to the method developed by Corman et al.^14^ until May 15 in Labor 28, Berlin, and thereafter in the State Laboratory of Berlin-Brandenburg (LLBB).

### Statistical analysis

Descriptive statistics were obtained on the characteristics of those infected. Risk ratios and Chi-square tests were performed to identify differences between female and male residents and staff in terms of infection and death. Moreover, the same methods were used to calculate excess mortality in NH01.a in March and April 2020. Microsoft Excel 2016 and R version 4.0.3 were used for the calculations.

## Results

### Infection and mortality rates of Nursing homes

A total of 946 individuals were affected by the three outbreaks as residents (502 persons) or due to their occupation (444 persons). In addition, there were 12 relatives of NH01.a employees who tested positive. Including these family members, 87 people were infected with SARS-CoV-2 during the three outbreaks (Fig. 4). Of the entire 502 residents, 48 persons (9.6%) tested positive, with the highest infection rate at NH01.a (34.4%). In NH02, 2.2% and in NH03, 5.6% became infected with SARS-CoV-2. Sixteen residents, representing 33.3% of infected residents and 3.2% of all nursing home residents, died from COVID-19. Of the deaths caused by the COVID-19 outbreak, twelve occurred in NH01.a, four in NH03, and none in NH02. (Fig. 4). Of the total of 444 staff members, 27 (6.1%) tested positive. One of them, an 82 year old male volunteer died from COVID-19. Fourteen of the survivors required temporary hospitalization for severe disease progression.

### Entry event and measures of the outbreak in NH01.a

The outbreak in NH01.a was the first outbreak in a Neukölln nursing home. At that time, the pandemic in Germany was still in the early stages of the first wave. The initial situation of the outbreak in NH01.a was a bowling event for residents, organized by an 82 year old volunteer in March 2020, shortly before the first measures to contain the pandemic were ordered by the German government. Nine days later, the volunteer employee was hospitalized for a different reason than COVID-19. On admission, a routine test detected a SARS-CoV-2 infection. During the following days, the person developed a cough and pneumonia and died 12 days after admission (Fig. 1; Table 1).

**Table 1:** Overview of confirmed COVID-19 cases in nursing home NH01.a. Order by the onset of symptoms.

ID	Status	Age	Sex	Quarantine	Hospitalized	Deceased
1	Volunteer	82	M	Yes	Yes	Yes
2	Resident	87	F	Yes	Yes	No
3	Resident	80	F	Yes	Yes	No
4	Resident	88	F	Yes	Yes	No
5	Resident	86	F	Yes	Yes	No
6	Staff	47	F	Yes	No	No
7	Staff	51	F	Yes	Yes	No
8	Resident	73	M	No	No	Yes
9	Resident	95	F	No	No	Yes
10	Relative	50	F	Yes	No	No
11	Resident	98	F	Yes	Yes	Yes
12	Staff	42	F	Yes	Yes	No
13	Staff	65	F	Yes	No	No
14	Relative	13	F	Yes	Yes	No
15	Relative	14	F	Yes	No	No
16	Resident	87	F	Yes	No	No
17	Staff	41	F	Yes	Yes	No
18	Staff	58	F	Yes	No	No
19	Resident	98	F	Yes	Yes	Yes
20	Resident	95	F	Yes	No	No
21	Resident	59	F	Yes	No	No
22	Resident	79	F	Yes	No	Yes
23	Resident	78	M	Yes	Yes	Yes
24	Resident	75	F	Yes	No	No
25	Resident	85	F	Yes	No	Yes
26	Resident	88	F	Yes	No	No
27	Resident	95	F	Yes	No	No
28	Resident	80	M	Yes	No	No
29	Staff	20	F	Yes	No	No
30	Relative	16	F	Yes	No	No
31	Relative	44	M	Yes	No	No
32	Resident	96	F	Yes	No	No
33	Resident	83	F	Yes	No	Yes
34	Resident	91	F	Yes	No	No
35	Resident	79	F	Yes	No	No
36	Resident	75	F	Yes	Yes	Yes
37	Resident	87	F	Yes	No	No
38	Resident	86	M	Yes	No	Yes
39	Relative	12	M	Yes	No	No
40	Staff	54	F	Yes	No	No
41	Staff	53	F	Yes	Yes	No
42	Staff	61	F	Yes	Yes	No
43	Relative	64	M	Yes	No	No
44	Relative	54	F	Yes	No	No
45	Staff	30	F	Yes	No	No
46	Staff	60	M	Yes	Yes	No
47	Staff	45	F	Yes	No	No
48	Resident	87	F	Yes	No	No
49	Resident	94	F	Yes	No	No
50	Resident	93	M	Yes	No	Yes
51	Resident	89	F	Yes	No	Yes
52	Staff	41	F	Yes	No	No
53	Relative	46	M	Yes	No	No
54	Relative	18	F	Yes	No	No
55	Resident	91	F	Yes	No	No
56	Resident	73	F	Yes	Yes	No
57	Relative	70	F	Yes	No	No
58	Staff	46	M	Yes	No	No
59	Relative	70	F	Yes	No	No
60	Staff	21	M	Yes	No	No
61	Staff	35	F	Yes	No	No

The day after the admission, the positive test result was reported to the Department of Public Health Neukölln. Immediate measures taken by the pandemic squad were the tracing of the infected person's contacts of the past 2 weeks and interventions in close cooperation with the management of the nursing home: the three floors of the nursing home were separated from each other. For the first and third floors, a temporary ban on admission and on visiting was imposed. Staff members who had contact with the index person in the previous 14 days were quarantined. Moreover, staff was equipped with PPE. RT-PCR tests for SARS-CoV-2 of residents and employees were carried out on site by a mobile swab team operated by the pandemic squad. Ninety residents and 79 staff members underwent testing over the span of 1 week. Of these, eighteen residents and four staff members received positive PCR test results. During that week, two residents died of COVID-19. Four further diseased residents and one employee had to be referred to hospital due to the symptoms they developed, such as cough, fever, general feeling of illness, and pneumonia. Since the residents living on the second floor were then also affected, the measures described above were extended to the second floor (Fig. 1).

RT-PCR tests for residents and staff members were conducted weekly on site (Fig. 1). After seven more residents were tested positive in the second screening and two further residents died as a result of a SARS-CoV-2 infection, the measures were intensified: the second floor was set up for confirmed cases, and the staff members of the different wards were strictly separated from each other. Quarantine of nurses was declared in case of confirmed infection. Employees without specific COVID-19 symptoms were tested frequently and allowed to work under strict hygiene standards. In order to meet the stricter hygiene standards, all employees received further training based on the guidelines of the Robert Koch Institute (RKI). For this purpose, the management created brief videos with low-threshold explanations for the staff.

### Characterization of the outbreak in NH01.a

It turned out that the index person was an 82 year old man with a pre-existing lung disease, who voluntarily worked in the nursing home. During a period of 5 weeks, a total of 61 persons tested positive for SARS-CoV-2 in the context of this outbreak (Fig. 4). The youngest patient was 12, the oldest 99 years old. The median age of all diseased persons was 73 years. Fourty-four (62.0%) persons with confirmed SARS-CoV-2 infection were female. Thirty-one (50.8%) of the diseased persons were residents, 18 (29.5%) worked in the nursing home, and 12 (19.7%) were relatives of infected staff members. None of the residents in the connected home NH01.b became infected. Of the infected, nine (52.9%) residents, seven (41.2%) staff members and one (5.9%) relative of an infected staff member were transferred to hospital. The voluntary staff member and twelve of the ninety residents (13.3%) died from the SARS-CoV-2 infection (Fig. 4).

**Figure 5:**
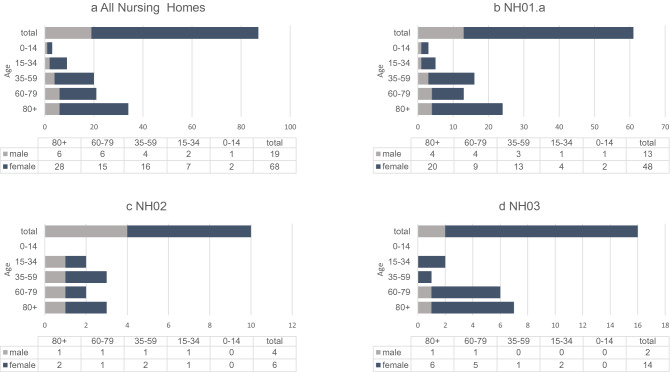
Overview of age and sex distribution among COVID-19 confirmed cases connected with outbreaks in three nursing homes. (**a**) Age and sex distribution of confirmed COVID-19 cases among residents and staff collected of the three nursing homes NH.01a, NH02, and NH03. (**b**) Age and sex distribution of confirmed COVID-19 cases among residents and staff of the nursing home NH.01a. (**c**) Age and sex distribution of confirmed COVID-19 cases among residents and staff of the nursing home NH02. (**d**) Age and sex distribution of confirmed COVID-19 cases among residents and staff of the nursing home NH03. Data are given as n numbers of each age cohorte.

**Figure 6:**
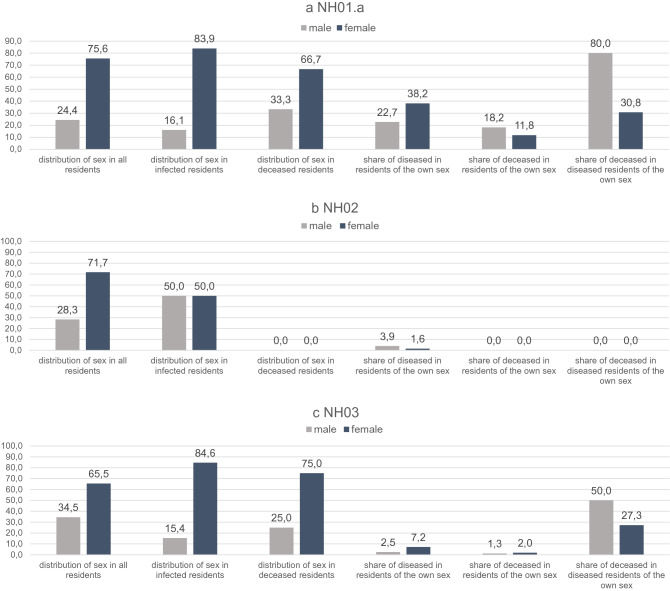
Sex distribution of diseased and deceased residents of the affected nursing homes. (**a**) Residents of NH01.a. Data are given in (%). (**b**) Residents of NH02. Data are given in (%). (**c**) Residents of NH03. Data are given in (%).

In total, 34.4% of the 90 residents and 16.7% of the care and service workforce were infected. Twelve relatives of staff members also developed a confirmed COVID-19 disease. Distribution of age and sex of all affected and non-affected persons is shown in Figs. 4, 5b and 6a. Of the 31 diseased residents, 83.9% were female. Considering that 75.6% of all residents were female, men were infected less often than women. Among the infected male residents, 80.0% died, while the mortality rate among the female residents was much lower, at 30.8% (Fig. 6a).

Relatives of nursing home residents were not affected as nursing home visitation restrictions were already enforced statewide before the first case in NH01.a occurred.

### Directed actions to prevent the spread to the connected nursing home NH01.b

In addition to the measures described, precautions were taken to prevent transmission of the virus to the connected nursing home. Since cooking is done in the kitchen of the affected home for NH01.a, NH01.b and two more locations, a personnel hygiene lock was installed there. It ensured to prevent contamination of the kitchen by hand and sole disinfection.

Furthermore, a problem arose when three residents of the affected home did not comply with the quarantine requirements, due to cognitive limitations. They continued to meet with residents of the neighboring home. In close cooperation between the home management and the Public Health Department Neukölln, a judicial quarantine was ordered for these three persons in accordance with the Infection Protection Act^11^ and implemented under the supervision of a caring person.

The entire staff and residents of NH01.b were tested for SARS-CoV-2 in a weekly interval for 4 weeks during the outbreak. No one in this nursing home got infected during the outbreak.

### Entry events and measures in the nursing homes NH02 and NH03

In NH02 and NH03, the virus was also introduced by staff. In NH02, the index was a 60 year old female caregiver (Table 2). Measures as described for NH01.a were taken immediately after reporting the test result to the pandemic squad of the Department of Public Health Neukölln (Fig. 2). During the following 7 days, four caregivers, one cleaning worker, and four residents tested positive. Distribution of age and sex of all affected persons is shown in Figs. 4, 5c, and 6b. Due to the immediate measures ordered by the pandemic squad of the Department of Public Health Neukölln, the outbreak could be limited to two of the six residential groups. None of the infected persons died.

**Table 2:** Overview of confirmed COVID-19 cases in nursing home NH02. Order by the onset of symptoms.

ID	Status	Age	Sex	Quarantine	Hospitalized	Deceased
1	Staff (index)	60	W	Yes	No	No
2	Staff	53	M	Yes	No	No
3	Staff	31	M	Yes	No	No
4	Staff	58	W	Yes	No	No
5	Staff	30	W	Yes	No	No
6	Resident	90	W	Yes	Unknown	No
7	Resident	91	W	Yes	Unknown	No
8	Resident	64	M	Yes	No	No
9	Staff	54	W	Yes	No	No
10	Resident	83	M	Yes	No	No

The index of the outbreak in NH03 was a 51 year old female nurse (Table 3). When her positive test result was reported to the pandemic squad, the same measures as described above for NH01.a were ordered by the Department of Public Health Neukölln (Fig. 3). In the following three and half weeks, all employees and residents were tested several times. In total, 13 residents and three staff members tested positive for SARS-CoV-2. Here, too, strict adherence to the measures succeeded in limiting the outbreak to two residential groups. When one resident in the second residential group tested positive, he was transferred to the area already affected, successfully preventing further spread in the second residential group. Nevertheless, one male and three female residents died of COVID-19 during the following 23 days (Fig. 3). More details about sex and age distribution of all non-infected, infected, and deceased residents and staff members are shown in Figs. 4, 5d, and 6c.

**Table 3:** Overview of confirmed COVID-19 cases in nursing home NH03. Order by the onset of symptoms.

ID	Status	Age	Sex	Quarantine	Hospitalized	Deceased
1	Staff (index)	51	W	Yes	No	No
2	Resident	66	W	No	No	Yes
3	Resident	79	W	Yes	No	No
4	Resident	68	W	Yes	No	No
5	Resident	79	W	Yes	No	No
6	Staff	31	W	Yes	No	No
7	Resident	97	W	Yes	No	No
8	Resident	76	W	Yes	No	No
9	Resident	91	M	Yes	Yes	Yes
10	Resident	99	W	Yes	No	No
11	Resident	90	W	Yes	No	No
12	Resident	60	M	Yes	Yes	No
13	Staff	25	M	Yes	Yes	No
14	Resident	81	W	Yes	No	No
15	Resident	90	W	Yes	No	Yes
16	Resident	93	W	Yes	No	Yes

### The influence of age and sex on infection and death

**Figure 7:**
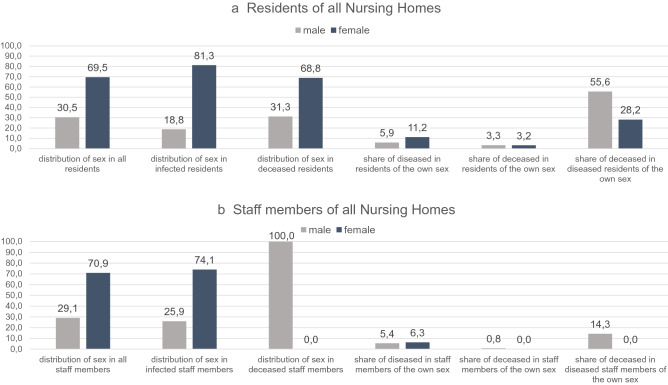
Sex distribution of diseased residents and staff members. (**a**) Residents of all three nursing homes. Data are given in (%). (**b**) Staff of all three nursing homes. Data are given in (%).

**Figure 8:**
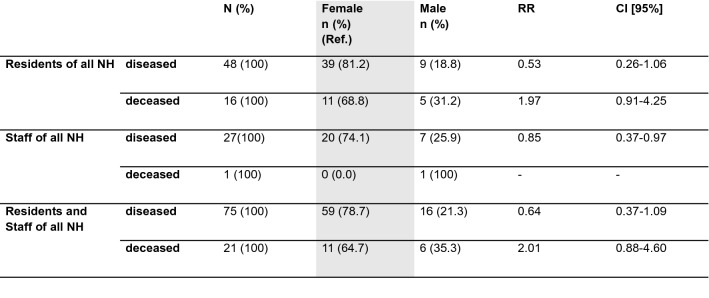
Significance of sex differences between the diseased and deceased residents and staff members of all affected nursing homes. Data are given in n-numbers (%) and risk ratios (RR) with 95% confidence interval.

The age and sex distribution among COVID-19 confirmed cases connected with the outbreaks are shown in Fig. 5a and 7. 43 (89.6%) of infected residents were at least 70 years old. Women had a higher risk of SARS-CoV-2 infection in all outbreaks described (Figs. 6 and 7), but had much lower mortality rates compared to men (Fig. 8). With respect to staff and residents of all nursing homes, men had a significantly lower risk of SARS-CoV-2 infection (RR 0.64, not significant), but a twofold increased risk of death if infected, although not significant (Fig. 8).

**Figure 9:**
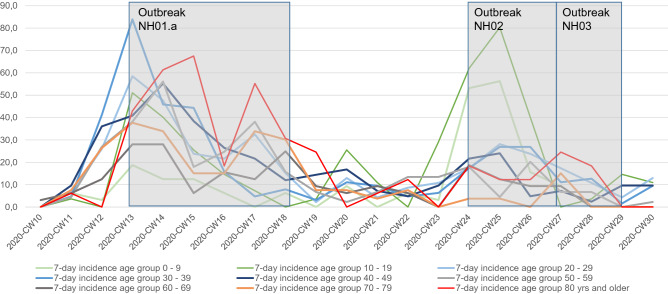
Age specific 7-days incidence in Berlin Neukölln. Data are given in RT-PCR confirmed cases of the last 7 days per 100,000 of age group.

**Figure 10:**
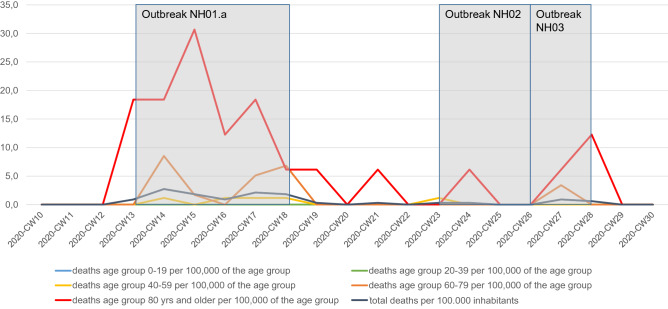
COVID-19-related deaths by age group in Berlin Neukölln. Data are given in deaths per calendar week per 100,000 of age group.

The large outbreak in NH01.a had a strong impact on the overall Neukölln data on age-specific incidence and age-specific COVID-19 death rates. The outbreak is reflected in an increased 7-day-incidence of 67.5 per 100,000 in the Neukölln population aged 80 and above (Fig. 9), and in the COVID-19 related mortality rate of 30.7 per 100,000 inhabitants of the same age group (Fig. 10).

### Excess mortality

**Figure 11:**
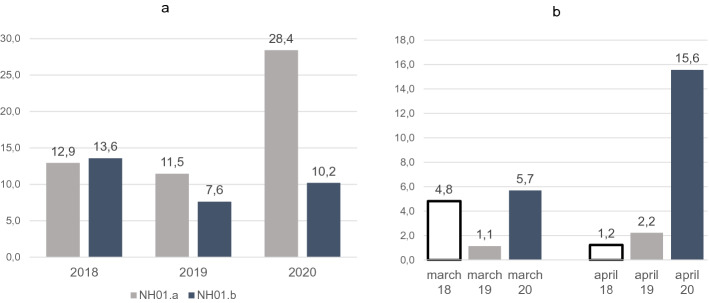
Mortality analysis among residents from 2018 until 2020. (**a**) Mortality among residents of NH01.a and NH01.b from 01/01 to 30/04 of the years 2018–2020. Data are given in (%). (**b**) Mortality of NH01.A residents in March and April for the years 2018, 2019 and 2020. Data are given in (%).

**Figure 12:**
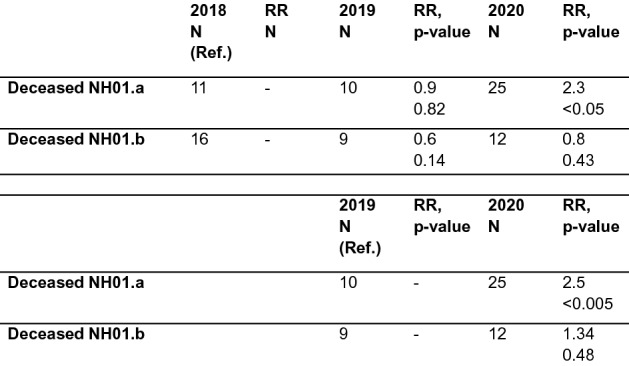
Excess Mortality among residents of the nursing home NH01.a and NH01.b from 01/01 to 10/5 of the years 2018–2020. Data are given in n-numbers and Risk Ratios (RR). Differences in infection numbers by year were tested for significance using a Chi-square test.

We compared the mortality rate of NH01.a from the two previous years to the rate during the outbreak, and found a clear excess mortality (Figs. 11, 12). The mortality rate in March 2020 was 1.2 times higher than in March 2018 and 5.2 times higher than in March 2019. In April 2020, 7.1 times as many residents died than in April 2018 and 13 times as many as in April 2019 (Fig. 11a,b). Comparing the mortality rates in 2020 from January 1st until May 10th, with those of the same period in the 2 years before, the mortality rate in 2020 for the nursing home affected is significantly higher. During this period, 27.8% of the residents died in 2020, 11.1% in 2019 (RR 2.5, p < 0.005) and 12.2% in 2018 (RR 2.3, p < 0.05). Mortality in 2018 and 2019 during this period did not differ significantly (Fig. 12). Neighboring NH01.b also showed no significant differences in mortality rates between past years and 2020 (Fig. 12).

## Discussion

In Germany, health policy is mainly operated through the federal states and their communities. During the pandemic, the local health departments play a key role in prevention of outbreaks and the containment of infection chains. They are in direct contact with the population and the management of facilities such as nursing homes, as well as with the federal state governments.

After the Department of Public Health Neukölln was informed that a volunteer worker of NH01.a tested positive for SARS-CoV-2, the first containment measures were implemented in close consultation with the management of the nursing home. Here, the good cooperation and close communication between the management and the health authority proved to be essential for successful containment strategies. Nevertheless, a total of 61 people got infected in connection with this outbreak, thirteen of them died. So, the question arises which lessons can be learnt. Especially, which measures have proven themselves to be helpful, which decisions should have been made differently and which factors make outbreaks in nursing homes so difficult to control.

**Figure 13:**
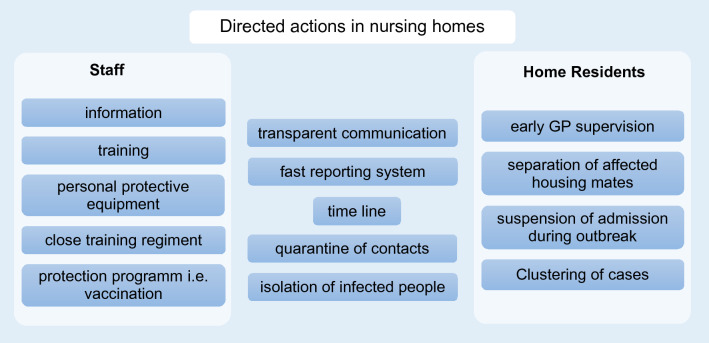
Directed actions in nursing homes in the event of an outbreak. Summary of our comparative analysis of the impact of actions in three different nursing homes. The conclusions indicate the area of conflicts between staff, home residents and the local public health department.

Nursing homes are among the most vulnerable institutions during the COVID-19 pandemic when vaccination programs were not implemented. A large proportion of deaths from COVID-19 are related to outbreaks in nursing homes^15^. This is due to shared accommodation, common activities and the demographic composition of their residents: they often are frail or suffer from pre-existing conditions, which is associated with severe disease progression^1,16,17^. Depending on the degree of care required, close physical contact with caregivers is inevitable. In the first outbreak in a Neukölln nursing home, 13.3% of all NH01.a residents died from COVID-19. Other outbreaks in nursing homes report an even higher mortality: Graham et al. report a mortality rate of 26% among residents in four British nursing homes^18^. In two nursing homes in King County, USA, 26% and 27.2% of the residents died in March 2020^19,20^. Based on the experience with the outbreak at NH01.a, outbreaks at NH02 and NH03 were contained more quickly, even though these two homes had at least twice as many residents as NH01.a. Measures that proved effective in the outbreaks described are shown in Fig. 13.

Compared to the two previous years, mortality during the outbreak at NH01.a was significantly higher. No significant differences in mortality between the relevant periods of 2018, 2019 and 2020 can be observed in NH01.b. Thus, it can be assumed that excess mortality in NH01.a is due to the COVID-19 outbreak. The infected male residents, in particular, showed a high mortality rate, in NH01.a their mortality rate was at 80.0% and in NH03 at 50%; both higher than that of infected female residents. In regard to all residents and all staff of the three homes, men had a lower risk of infection (RR 0.64, not significant), but their risk of death in case of infection was twice as high as that of women (RR 2.01, not significant) (Fig. 8). Due to the small number of cases no conclusion can be drawn from our analysis for a generally higher mortality. Nevertheless, since Graham et al. also report a significantly higher mortality risk in men in their analysis of three hundred and ninety-four SARS-CoV-2 positive nursing home residents, these data can be interpreted as indicating greater vulnerability among older men^18^.

SARS-CoV-2 is difficult to contain because, among other reasons, asymptomatic and pre-symptomatic infected persons can already spread the virus^21^. When investigating an outbreak in a nursing home in King County, USA, Kimball et al. identified 23 residents with positive RT-PCR tests^22^. Of these, only ten (43%) had symptoms: eight had symptoms typical of COVID-19 and two had atypical symptoms. However, 13 (57%) of the residents tested positive for SARS-CoV-2 were asymptomatic at the time of the swab. Of these, ten residents developed symptoms in the following 7 days^22^. In another nursing home in the same county, the situation appeared similar: of the residents who tested positive for SARS-CoV-2, 35% had specific symptoms, 8% had non-specific symptoms, and 56% were asymptomatic^19^. In the UK, 65.2% of 126 nursing home residents who tested positive developed no symptoms, while a further 19.8% showed atypical symptoms such as confusion, anorexia and gastrointestinal complaints^18^. According to Stall et al.^17^, frail people are more likely to show atypical symptoms such as delirium, falls, and function decline. Based on the knowledge that symptoms can be absent or atypical despite contagiousness, the aforementioned outbreaks were handled in such a way that all residents with direct contact to diseased or positively tested persons were quarantined by the public health department until a negative PCR test result was obtained. In addition, new admissions and visits by relatives were temporarily suspended when the first infection became known. Residents and staff members were tested weekly for SARS-CoV-2. Due to the spatial and organizational interdependence with NH01.a, residents and staff members of NH01.b were also tested three times at intervals of about 10 days. The immediate separation of NH01.a and NH01.b proved to be a successful strategy to protect NH01.b from the introduction of the virus. The separation of the two nursing homes was accompanied by spatial and organizational measures. On the spatial level, a hygiene lock was installed in the kitchen in NH01.a, which is used for the preparation of food in both (and two additional) facilities.

It is of great importance to train not only the nursing staff sufficiently, but also the service personnel, and to make sure that employees with other first languages get the essential information according to their language skills. Therefore, like the nursing staff, the service staff was also trained in extended hygiene measures and informed by corresponding information material. The latter included brief videos in order to inform international employees who did not speak the German language sufficiently.

### Directed actions and stakeholder compliance

All these measures, especially testing, physical distance and quarantine, require compliance from all parties involved. Compliance was good for most residents and staff members. The training and information materials for the staff members, as well as transparent communication with the residents and their families by the facility managements were important. However, in the case of the outbreak in NH01.a it was problematic to enforce contact restrictions with the neighboring nursing home for those residents who could hardly be swayed by reason due to cognitive limitations. This was the case for three residents of NH01.a with positive test results and one resident of NH01.b who had direct contact to infected residents of NH01.b. Although restrictions on freedom are a fundamental encroachment on personal rights, which are anchored in the German constitution, a court order based on the Infection Protection Act was issued to protect the residents of NH01.b^11,23^. This provided that the three persons concerned, were allowed to be detained in their living quarters against their free will. In the case of such coercive measures, it is of great importance that adequate care is provided for the detained persons. This is especially true for people with cognitive disabilities such as dementia, for whom isolation in a room can be incomprehensible and very stressful^22^. In the case described here, the persons concerned received intensive care from additional nursing staff during the quarantine. Due to the duration of the judicial assessment of the situation, 8 days elapsed from the time of positive test results, in which the persons concerned did not comply with the quarantine regulations. Even if in this particular case the virus was not introduced into NH01.b as a result of this delay, the lesson learned of this experience is that it is important to draw up a plan in advance of an outbreak, defining and outlining measures to be taken in such cases, in order not to lose valuable time in case of an emergency.

Healthcare professionals caring for COVID-19 patients are at increased risk of self-infecting and spreading the infection to other parts of the facility or to their private environment^13^. Several measures have been taken to prevent the infection of the healthcare staff. The RKI recommends not only to isolate the infected, but to divide the affected nursing home into an infectious and a non-infectious area^13^. In the affected nursing homes described here, this was done by having some of the residents change rooms and one area reserved for infected residents who were cared for by the same pool of nursing staff. In NH01.a, this happened two and a half weeks after the infection of the index case became known. In retrospect, considering the high number of infected residents and nursing staff, it seems possible that the decision to take this measure should have been taken earlier, as was accordingly done in the subsequent outbreaks at NH02 and NH03.

To avoid exposing too many physicians to the risk of infection when dealing with infected home residents, a collaboration with a nearby general practitioner who cared for all cases in the nursing home during the outbreaks proved effective.

Furthermore, it is of great importance that PPE is available to the staff in situations of contact with infected residents. Therefore, one of the first measures taken by the public health department was to equip the nursing staff with PPE, which was lacking due to supply bottlenecks at the beginning of the pandemic in Germany. However, experiences from a US study showed that even trained nursing staff is not fundamentally experienced in the correct use of PPE^22^. Because this was also found in NH01.a, the first outbreak in Neukölln, the management of the nursing home carried out training courses on the extended hygiene measures with the staff.

With regard to contact person management, the department of public health Neukölln followed the guidelines of the RKI. These distinguished three categories of contact persons depending on the duration and intensity of contact with the infected person. Nursing staff of COVID-19 patients without sufficient equipment with PPE are considered contacts of the first category and are quarantined. However, nurses who are adequately equipped with PPE and who use PPE correctly belong to category III and may work under safety precautions^13^. This is of great importance to maintain the functioning of the nursing homes, especially if a part of the nursing staff is already infected and absent for a longer period of time^15^. In exceptional cases, however, quarantine was imposed on asymptomic nurses until a negative PCR test result was obtained. These nurses lived in other districts than Neukölln. Since the federal structures in Germany mean that other public health departments are responsible for these districts, measures are not consistent. In order to compensate for the lack of staff due to quarantined and diseased nurses, leased employees were hired. Since sufficient and well-trained staff is important to ensure the functioning of the nursing home and to keep the number of infections as low as possible, it is important to employ such additional staff for a longer period of time.

In retrospect, the following measures can therefore be assessed positively: It was important to have transparent communication and cooperation between the public health department, the nursing home’s management, the service and nursing staff, the residents and their relatives. This included a flow of information and staff training. Immediate measures such as isolation of the infected, quarantining of contacts among the residents, equipping the staff with PPE and close-meshed testing instead of quarantining nursing staff proved to be effective in fighting the virus while maintaining services. Particularly in the case of pathogens which, like SARS-CoV-2, are unknown or little researched to this date, it is important that the scientific findings are continuously observed, especially by the health authorities, and that measures are adapted flexibly to the current state of knowledge.

However, in order not to waste valuable time, pandemic plans should be drawn up in advance by the nursing homes in cooperation with the local health authorities, which also take into account how to deal with cognitively impaired residents. In addition, a structured documentation system is needed in the health authorities in which all data relevant for containing the outbreak, such as test results and measures taken, are systematically stored and accessible to the staff of the pandemic team. The described outbreak took place at an early stage in the first wave of the pandemic in Germany 2020, so that pandemic teams had to be newly formed and new structures had to be established. As a result, the many people initially involved used different systems for storing data before systematizing it, which can lead to incomplete or incorrect documentation. A faster reporting system that allows coordination with neighboring districts is also lacking in Berlin to date. A backlog demand can be observed here, which is due in particular to the fact that health authorities in Germany have been poorly equipped financially and in terms of personnel in recent years. This has had a negative impact on the degree of digitization, among other things^24^. The same applies to nursing homes, which are often understaffed because many vacancies cannot be staffed due to skills shortages. Hygienic work in nursing requires time, as Stahmeyer et al. have demonstrated, using the example of a German intensive care unit^25^. For this reason, a health care system must ensure that care and health facilities can be adequately staffed. These are overarching measures that cannot be solved in the short term, but whose importance become obvious during the pandemic. In Neukölln, during the pandemic, an impending shortage of personnel in facilities is recorded at 2-week intervals by the public health department in order to be able to intervene in time. Through a platform initiated by the Berlin government, nursing staff can be found in situations of bottlenecks^26^. This aims to reduce the risk of nosocomial infections caused by overworked healthcare workers. In order to avoid shortages in supply, it can also be useful to pursue previously uncommon approaches. In Toronto, Canada, a partnership between an affected nursing home and a nearby hospital has proven successful. The hospital has agreed in advance to provide personnel, equipment and clinical expertise in case of an outbreak^17^. Furthermore, nursing homes and health authorities should decide on temporary restrictions for visits and community activities depending on the local infection situation. Kimball et al. also recommend the wearing of face masks by nursing home residents and regular cohort testing among residents and caregivers^22^. However, the latter should be discussed in the context of testing capacity and regional infection rates, as low prevalence increases the percentage of false positives^27^. This has to be considered especially in cases where the rapid antigen point-of-care tests are in use. As of September 2020, 47.1% of COVID-19 cases in Neukölln, are connected to outbreaks in nursing homes. However, there is an observable trend that fewer nursing homes are affected by outbreaks during the ongoing pandemic. This can be interpreted as a sign that lessons have been quickly learned from the experience of the first outbreaks and that preventive measures as well as the vaccination program are successful.

This report describes an outbreak in a nursing home under its specific conditions. Therefore, the experiences cannot be fully transferred to other outbreak events. However, our analysis can solely give an impression of what directive measures could be effective and what aspects should be considered when deciding on measures. Due to the small number of cases, the information value of the statistical analyses is limited. So far there are few studies on COVID-19 outbreaks in nursing homes. To substantiate these statements, evaluations of data from a larger number of nursing homes would be necessary.

## Supplementary Information

Supplementary Legends.

Supplementary Figure S1.

## Abbreviations

COVID-19: Corona virus disease 2019
NH: Nursing Home
PPE: Personal protective equipment
RKI: Robert Koch Institute, the national public health institute of Germany
RT-PCR: Reverse transcription-polymerase chain reaction
SARS-CoV-2: Severe Acute Respiratory Syndrome Coronavirus 2
WHO: World Health Organization
