# Supplementary material for: Characterization of COVID-19 outbreaks in three nursing homes during the first wave in Berlin, Germany
Source: Sci Rep. 2021 Dec 24;11:24441. doi: 10.1038/s41598-021-04115-9 (PMC8709844; doi:10.1038/s41598-021-04115-9)
Supplement: Supplementary file 1 — Supplementary Legends. [file 41598_2021_4115_MOESM1_ESM.docx]

**Characterization of COVID-19 outbreaks in three nursing homes during the first wave in Berlin, Germany**

*Alexandra Roth^a,b, *^, Silke Feller^a^, Andreas Ruhnau^a^, Lena Plamp^a^, Ute Viereck^c^, Kerstin Weber^a^, Dominic Maertens^a^, Ilona Hoor^a^, Ronny Gamradt^a^, Pia Freyer^a^, Frank Wenke-Gellert^a^, Andreas Terjaew^a^, Andreas Zintel^a^, Juliane Markus^a^, Ines Gögelein-Mahfouz^a^ and Nicolai Savaskan^a,*^*

^a Department of Public Health Neukölln, District Office Neukölln of Berlin Neukölln, Berlin, Germany;^

^b University of Applied Sciences, Fulda, Germany;^

^c Department of Legal Office Neukölln, District Office of Berlin Neukölln, Berlin, Germany^

**Supplement Legend**

**Figure S1. Sex distribution of diseased residents and staff members of all nursing homes.**

Data are given in (%).
