# Supplementary figures and images for: Characterization of COVID-19 outbreaks in three nursing homes during the first wave in Berlin, Germany
Source: Sci Rep. 2021 Dec 24;11:24441. doi: 10.1038/s41598-021-04115-9 (PMC8709844; doi:10.1038/s41598-021-04115-9)

Roth et al. Fig. 14 Sex distribution of diseased residents and staff members of all nursing homes [%].

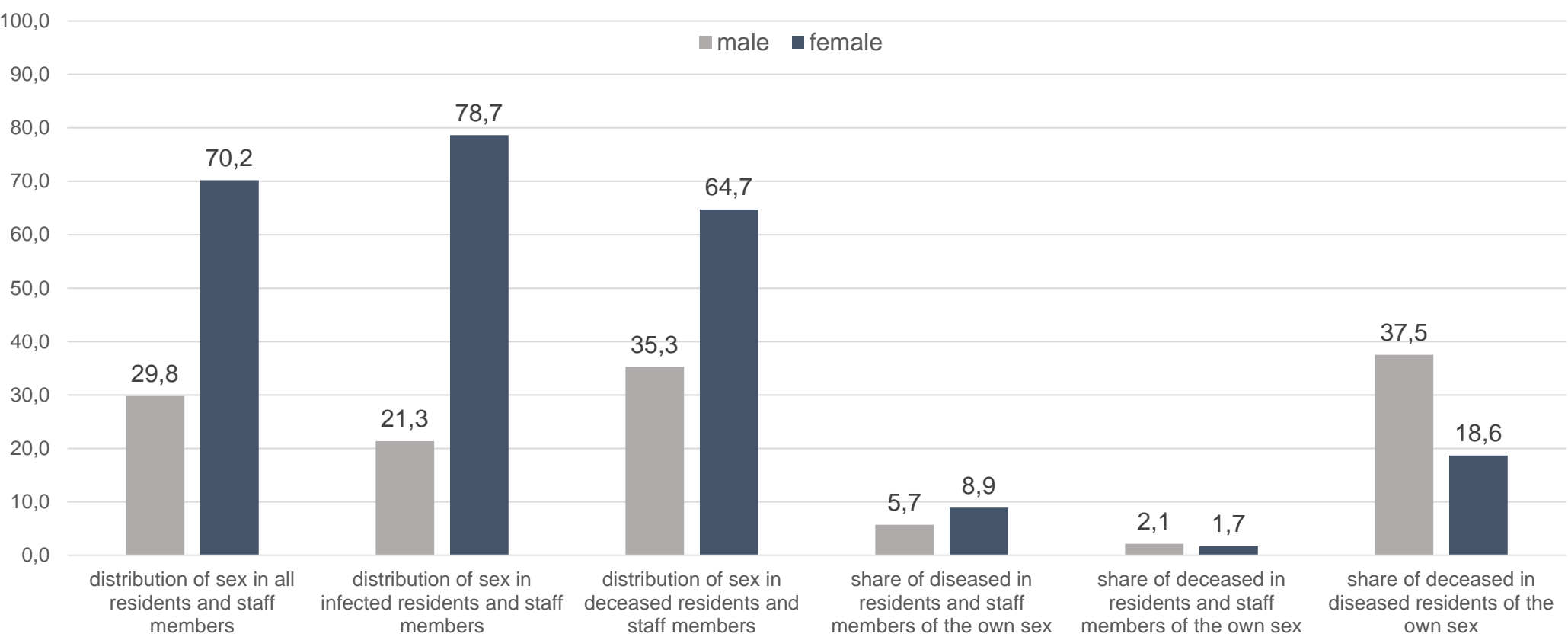

Supplement: Supplementary file 2 — Supplementary Figure S1. [file 41598_2021_4115_MOESM2_ESM.pdf]
